# A New Computational Model for Astrocytes and Their Role in Biologically Realistic Neural Networks

**DOI:** 10.1155/2018/3689487

**Published:** 2018-07-05

**Authors:** Zahra Sajedinia, Sébastien Hélie

**Affiliations:** Department of Psychological Sciences, Purdue University, West Lafayette, IN 47907, USA

## Abstract

Recent studies in neuroscience show that astrocytes alongside neurons participate in modulating synapses. It led to the new concept of “tripartite synapse”, which means that a synapse consists of three parts: presynaptic neuron, postsynaptic neuron, and neighboring astrocytes. However, it is still unclear what role is played by the astrocytes in the tripartite synapse. Detailed biocomputational modeling may help generate testable hypotheses. In this article, we aim to study the role of astrocytes in synaptic plasticity by exploring whether tripartite synapses are capable of improving the performance of a neural network. To achieve this goal, we developed a computational model of astrocytes based on the Izhikevich simple model of neurons. Next, two neural networks were implemented. The first network was only composed of neurons and had standard bipartite synapses. The second network included both neurons and astrocytes and had tripartite synapses. We used reinforcement learning and tested the networks on categorizing random stimuli. The results show that tripartite synapses are able to improve the performance of a neural network and lead to higher accuracy in a classification task. However, the bipartite network was more robust to noise. This research provides computational evidence to begin elucidating the possible beneficial role of astrocytes in synaptic plasticity and performance of a neural network.

## 1. Introduction

Neurons and glia cells are building blocks of the human brain. Neurons are defined based on their ability to produce action potentials; the other cells in the human brain, which do not support this ability, are called glia cells [1]. By the early 1990s, it was widely believed that glia cells only performed passive functions, such as providing nutrition and removing waste. They were referred to as* housekeeping cells* [2, 3]. In 1999, for the first time, the term “tripartite synapse” was introduced by Araque et al. to describe the bidirectional communication between neurons and glia cells [4]. Since then, each year new evidence supports the hypothesis that glia cells, alongside neurons, communicate with synapses and modulate them [4–18]. One consequence of these findings is that glia cells are responsible for processing information in the human brain.

These findings are important because glia cells are up to 50 times more numerous than neurons [19]. They come in different shapes and at different locations in the nervous system [1]. So far, only two types of glia cells, named Schwann cells, in the neuromuscular junctions, and astrocytes, in the central nervous system (CNS), have been shown to be associated with synapses and participate in synaptic modulation [4, 18]. In this article, we focus on CNS tripartite synapses, and, therefore, we only consider astrocytes.

### 1.1. What Is the Role of Astrocytes in Neural Computation?

Given the mounting evidence that astrocytes contribute to neural computation, a follow–up question is what roles do astrocytes play in neural computation? One intriguing possibility is that astrocytes could contribute to learning and memory [20]. For example, astrocyte disruption impairs later formation of long-term memory. In addition, evidence has been gathered that astrocytes affect the dynamics of neural populations [21], which could modulate neural plasticity [22]. One possible explanation for these observations is that astrocytes can operate at slower timescales than neurons [23, 24] and thus could possibly maintain activity in postsynaptic neurons after stimulation of the presynaptic neurons has stopped. This in turn could facilitate consolidation by facilitating long-term potentiation (LTP) and long-term depression (LTD) [25]. However, another possibility is that this extended neural activity alongside other noise sources adds noise in the system, which could affect the network's robustness and performance [26]. One way to test for these possibilities is through computer simulation. Because this hypothesis is related to rhythms and timing, the present research used biologically realistic spiking neuron models and developed a dynamical model of astrocyte activation. Below we present minimum criteria that a dynamical model of astrocyte activation should meet and review previous attempts at computational models of astrocytes.

### 1.2. Previous Modeling Effort

Many computational neuroscience models of astrocytes have been proposed to account for the many differences between neurons and astrocytes [15]. However, in this project we propose a new approach by using an existing neural model to implement astrocytes, namely, the Izhikevich simple model of neurons [27]. An important advantage of the proposed approach is that it allows researchers to simply model astrocytes as a type of neuron, without sacrificing the two key characteristics of astrocyte dynamics. First, as suggested by the absence of action potentials, astrocytes show a linear current-voltage relationship (I–V curve) [28]. In contrast, the I–V curve is nonlinear in most neurons (and often N–shaped) [27]. Second, the effect of astrocyte modulating synapses can be slower than neurons, as studies show that astrocytes can be in a slow or fast mode. The slow and fast modes target NMDA and mGluR receptors, respectively [29]. In this article, we study the behavior of networks by using reinforcement learning rules. Since LTP in reinforcement learning relies on NMDA receptors [25], this article focuses on the slow mode of astrocyte dynamics [23, 24].

Note that many existing astrocyte models do not account for one or both these characteristics. For example, some existing models are not presenting a linear I–V curve in astrocytes [30, 31]. As a result, astrocytes spike in the model proposed by Haghiri et al. [32]. Other models focus on the tripartite synapse and are not capturing the intercellular characteristics of astrocytes [24, 33, 34]. In the present work, we attempt to propose a simple biologically realistic model of astrocyte dynamics that meets these minimum requirements.

### 1.3. Organization of This Article

This research aimed to study the role of astrocytes in the performance of neural networks. More specifically, we intended to test whether astrocytes are capable of improving the performance of a spiking neural network. The reminder of this article is organized as follows. First, Section 2 describes a new model for astrocytes by using the Izhikevich model of neurons. Second, Section 3 describes the design and implementation of two networks of neurons. The first network had only bipartite synapses and the second network also included astrocytes and thus tripartite synapses. These networks are referred to as* bipartite* and* tripartite* networks (respectively). Third, by applying reinforcement learning rules, we studied the classification accuracy of both networks in noisy and non-noisy conditions. The results in Section 3.2 show that in non-noisy environments, adding astrocytes can lead to a higher accuracy in classifying randomly generated stimuli. Lastly, Section 4 discusses the results and explores future directions for studying astrocytes in both healthy and nonhealthy brains as well as in artificial intelligence.

## 2. Cell Models

Although astrocytes recently received much attention in neurophysiology [35–38], their computational model remain underdeveloped when compared to their neural counterparts [15]. In this section, we introduce a new model of astrocytes based on the Izhikevich model of neurons [27]. The proposed model is aimed at reproducing the linear I–V curve observed in astrocytes [28]. The timing of tripartite synaptic modulation is addressed in Section 3.1.2.

### 2.1. Izhikevich's Simple Neuron Model

The Izhikevich model is a computationally efficient, biologically plausible, model of neurons that allows for real-time simulation of networks of spiking neurons on a desktop PC [39]. Each neuron in the Izhikevich model is implemented as follows [27]:

(1) Cv˙=kv−vrv−vt−u+Iu˙=abv−vr−uif  v≥vpeak,  then  v←c,  u←u+d

where *C* represents the membrane capacitance, *v* is the membrane potential, *v*_*r*_ is the resting membrane potential, *v*_*t*_ is the instantaneous spiking threshold, *I* is the input, *u* is the recovery current, and *a* is a recovery time constant. Rheobase and input resistance jointly determine the constants *k* and *b*. *c* and *d* represent the voltage reset value and the total difference between the outward currents and inward currents during a spike (respectively). These parameters can be set to different values to accurately model many types of neurons [27]. In this article, neurons were modeled by using the parameter values provided by Izhikevich to simulate cortical pyramidal neurons (Table 1).

### 2.2. Dynamic Model of Astrocyte Activation

**Figure 1:**
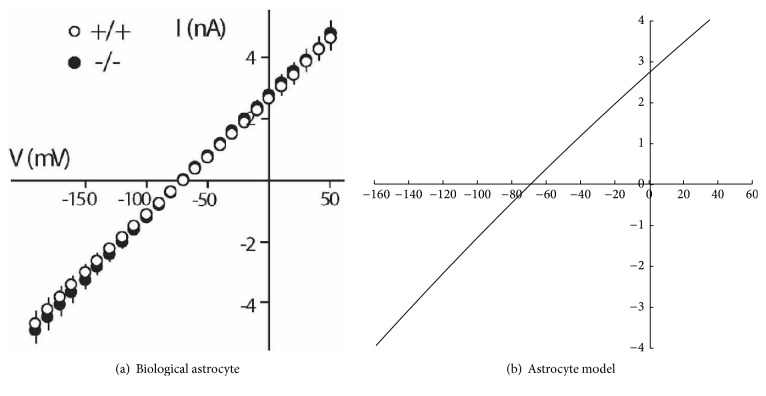
The current/voltage relationship (IV curve) for a biological astrocyte (a) Pannasch et al. [28] (Supplementary Material), the model (b).

The Izhikevich model of neurons is flexible in modeling different types of neurons. However, the Izhikevich model has never been used to model astrocytes, and no parameter values were previously available. As shown in Figure 1(a), the relation between voltage and current in astrocytes is approximately linear [28, 40]. We used the IV relationship in Figure 1(a) to estimate the parameters of the Izhikevich model that could emulate the astrocyte voltage-current curve. The parameters of the Izhikevich model were optimized (using mean square error) to values that give an approximate linear voltage-current relation. Table 1 shows the estimated values of an Izhikevich neuron that represent an astrocyte. It should be noted that the parameters in the astrocyte model do not have the same physiological interpretation as in the neuron model. The values of *C*, *v* and the other parameters were estimated to represent the linear I–V relation in astrocytes and they do not correspond to the astrocyte's capacitance, voltage, and so on. Also, *v* mostly represents *Na*^+^ in the Izhikevich model of neurons, whereas it represents *Ca*^2+^ in the proposed astrocyte model. Hence, the astrocyte model parameter values should be interpreted as scale–free.

### 2.3. Results

**Figure 2:**
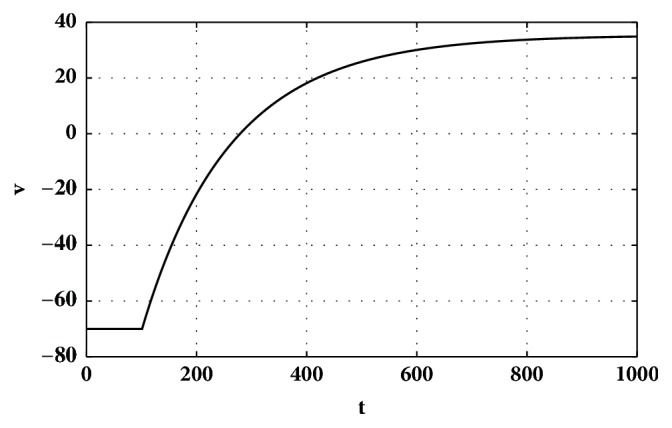
Membrane potential of the astrocyte model by injecting the current of I=4mA from t=100ms to t=1000ms.

Figure 1 shows the current/voltage (IV) curves of a biological astrocyte on the left and the new astrocyte model on the right. *r*^2^ between the data and model is 0.99, which indicates a near-perfect fit. Both curves show a linear relation between current and voltage. Also, Figure 2 shows the membrane potential of a simulated astrocyte with a stable input current of 4*mA* from *t* = 100*ms* to *t* = 1000*ms*. Biological astrocytes do not spike in these conditions [40]. Similarly, the astrocyte model did not produce any spike. Note, however, that injecting strong currents in the astrocyte model would eventually result in spikes. However, we did not have biological information on astrocyte behavior in those current ranges. Hence, the model is compatible with the available biological data in Dallérac et al. [40].

### 2.4. Discussion

This section proposed a new biologically realistic astrocyte model that accurately represent the linear IV relationship and does not spike. Given that the natural shape of the IV relationship in the Izhikevich model is nonlinear, the reader may wonder why we chose not to use a linear equation to model astrocytes instead of Izhikevich neuron's equations. Our choice was motivated by the fact that the Izhikevich model is popular and well-defined. Hence, the proposed model allows for modeling astrocytes simply by modifying the values of the parameters of available neurons. This makes the inclusion of astrocytes convenient in neural networks using the Izhikevich model, as astrocytes can be modeled as just another type of neurons.

The change in the membrane potential is studied by simulating the injection of the current of 4*mA* to the astrocyte model (Figure 2). The reason that we chose the 4*mA* current is that the biological results in Dallérac et al. [40] were based on the same condition. Therefore, to make the comparison possible, we kept the conditions equivalent. The results show that there are no spikes in both the biological and simulated astrocytes. The new astrocyte model thus satisfies the first key characteristic of biological astrocytes.

## 3. Modeling and Testing Bipartite and Tripartite Networks

Section 3.1 presents the design, implementation, and test procedure of the bipartite and tripartite networks. Then, results and discussion are provided in Sections 3.2 and 3.3, respectively.

### 3.1. Method

To study how astrocytes affect synaptic plasticity and the network's overall performance, we implemented two networks: the first network contained neurons and bipartite synapses (Section 3.1.1), while the second network contained neurons, astrocytes, and tripartite synapses (Section 3.1.2). Both of these networks were trained with reinforcement learning, as described in Section 3.1.3. Finally, Section 3.1.4 details the learning task implementation method for comparing the results.

#### 3.1.1. Bipartite Network

**Figure 3:**
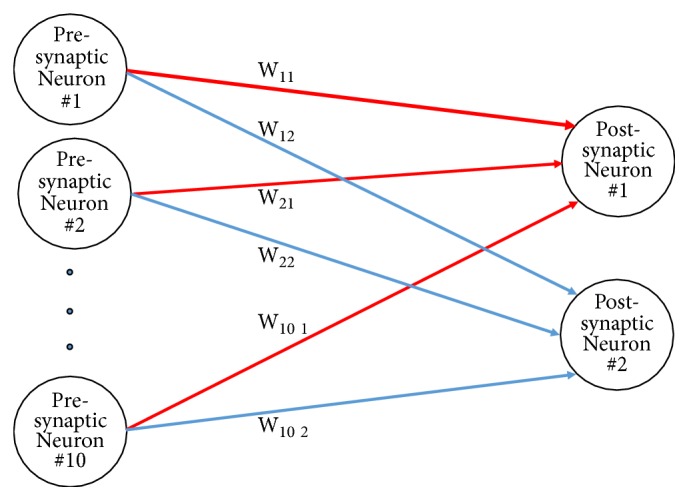
The architecture of the bipartite network. This model consists of 10 presynaptic neurons, 2 postsynaptic neurons, and 20 plastic synapses. *W*_*ij*_ represents the synaptic weight from presynaptic neuron *i* to postsynaptic neuron *j*.

*Architecture*. The network of neurons had 10 presynaptic neurons, 2 postsynaptic neurons, and 20 fully connected plastic synapses (Figure 3). The neurons were modeled based on the Izhikevich simple model of neurons, as described in Section 2.1.

*Modeling Synapses.* The simulated synapse can be simplified by modeling the delays of spike propagation through the synaptic cleft. One standard and widely accepted method is to use an *α*-function [25, 41]:

(2) $$\begin{array}{c} f\left(\;t\;\right)=\frac{t}{\lambda }\exp\left(\;\frac{\lambda -t}{\lambda }\;\right) \end{array}$$

where *t* is the time since the cell voltage reached *v*_*peak*_ and *t* = 0 is the time at which the cell voltage reached *v*_*peak*_. *λ* is a constant that determines the duration of signal propagation in the synapse. Greater *λ* values result in longer synaptic transmission. The *α*-function delivers the neurotransmitter from the presynaptic neuron to the postsynaptic neuron gradually. If *v*_*peak*_ is reached again by the presynaptic neuron or an astrocyte while the propagation of the neurotransmitter is still in process, then a new *α*-function related to reaching the second *v*_*peak*_ is added to the first *α*-function. The latency in a typical synapse is generally less than 0.5 ms [42]. This delay was approximated by using *λ* = 125.

#### 3.1.2. Tripartite Network

In the tripartite network, astrocytes were modeled as proposed in Section 2.2. Neurons were identical to the Izhikevich simple model of neurons, which are presented in Section 2.1. However, synapses were different and designed as follows.

**Figure 4:**
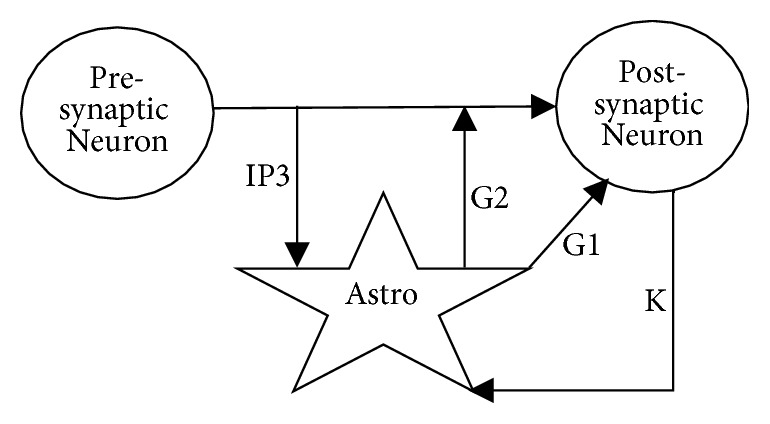
Simplified signaling pathways in a tripartite synapse. The astrocyte receives *IP*3 from the presynaptic neuron and *k*^+^ from the postsynaptic neuron. The astrocyte sends glutamate to the synapse and the postsynaptic neuron as shown by *G*1 and *G*2.

*Modeling Tripartite Synapses.* The process of synaptic neurotransmission is typically initiated by the release of neurotransmitters by the presynaptic neurons. These neurotransmitters can reach adjacent astrocytes and increase *Ca*^2+^ concentration inside the cell. This increase of *Ca*^2+^ can cause astrocytes to release glutamate. This glutamate then feeds back to the synapse and neurons [4].  Figure 4 shows a simplified model of this process.

To model the signaling pathways of *IP*3, *k*^+^ and glutamate, we used an *α*–function with *λ* = 1000 for astrocyte's glutamate and *IP*3 and *λ* = 100 for the *k*^+^ pathway. These values approximately reproduce the greater latency in tripartite synapses.

**Figure 5:**
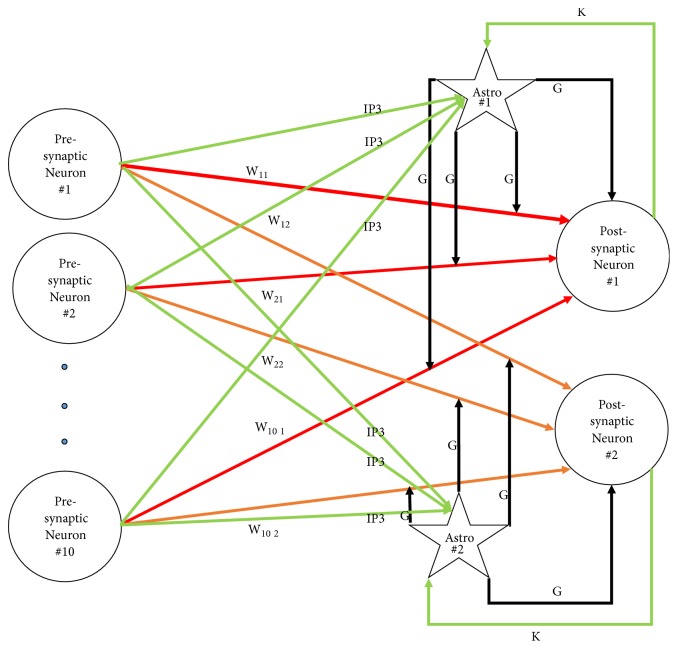
Architecture of the tripartite network. Astrocytes are shown as stars. The neurotransmitter associated with each synapse is indicated on top of each line. G stands for glutamate and k stands for *k*^+^. *W* represents the weight of a synapse. Red lines show the inputs to the first postsynaptic neuron; orange lines show the inputs to the second postsynaptic neuron. Green lines and black lines represent inputs to, and output from, astrocytes (respectively). Connections between neurons (red and orange) were plastic, while the rest of the connections were fixed. Connections with the same symbol had the same constant weight values.

*Architecture.* The architecture of the tripartite network is similar to the bipartite network except that, in addition to neurons, 2 astrocytes were included (one for each postsynaptic neuron) and the resulting synapses were tripartite. Astrocytes and their relation to synapses and neurons are depicted in Figure 5. As can be seen in the figure, each astrocyte contributed to 10 synapses and received input from all presynaptic neurons as well as its associated postsynaptic neuron.

#### 3.1.3. Synaptic Plasticity

Synaptic plasticity can be presented in terms of different learning models. In this research, we used the reinforcement learning algorithm described by [25]. In this model, LTP is triggered by (1) strong presynaptic activation, (2) strong postsynaptic activation, and (3) dopamine levels above baseline. In contrast, LTD is triggered by strong pre- and postsynaptic activation with dopamine below baseline or weak postsynaptic activation. This learning process is described by the following:

(3) wK,Jn+1=wK,Jn+αwIKnSJn−θNMDA+·Dn−Dbase+wmax−wK,Jn−βwIknSJn−θNMDA+·Dbase−Dn+wK,Jn−γwIKn·θNMDA−SJn+·SJn−θAMPA+wK,Jn

where *w*_*K*,*J*_(*n*) is the strength of the synapse on trial *n*. *I*_*k*_ represents the input from the presynaptic neuron (i.e., ∫*f*[*V*_*A*_(*t*)]*dt*, which is the integrated *α*-function output of the presynaptic neuron). *S*_*j*_ is the integral over the positive voltage of postsynaptic neuron *j*, *D*_*base*_ is a constant that shows the baseline dopamine level, *D*(*n*) denotes the amount of dopamine released following feedback on trial *n*, and *α*_*w*_, *β*_*w*_, and *γ*_*w*_ are constants that work similar to standard learning rates. *θ*_*NMDA*_ and *θ*_*AMPA*_ are the activation thresholds for postsynaptic *NMDA* and *AMPA* glutamate receptors (numerically *θ*_*NMDA*_ should be greater than *θ*_*AMPA*_ [25]). [*x*]^+^ represent a function that returns 0 for negative values and keeps the same value for positive values. Note that weights are not modified when the postsynaptic activation is below *θ*_*AMPA*_ (see last term of (3)). Finally, *w*_*max*_ is the maximum allowable weight.

To calculate *D*(*n*), we used the following formula:

(4) $$\begin{array}{c} D\left(\;n\;\right)=\left\{\begin{array}{cc} 1 & if\;\;RPE>1\\ 0.8\times RPE+0.2 & if\;\;-0.25\leq RPE\leq 1\\ 0, & if\;\;RPE<-0.25. \end{array}\right. \end{array}$$

where RPE is

(5) RPE=ObtainedRewardRn−PredictedRewardPn

Predicted reward, *P*_*n*_, is

(6) $$\begin{array}{c} P_{n+1}=P_{n}+\eta \left(\;R_{n}-P_{n}\;\right) \end{array}$$

Obtained Reward is +1 if the network is correct, −1 if the network is incorrect, and 0 if no feedback is received [25].  Table 2 shows the values assigned to the constant parameters of the above equations.

#### 3.1.4. Networks in Action and Comparisons

To test for the learning ability of the bipartite and tripartite networks, a simple classification experiment was designed. To generate classification stimuli, the input layer of the networks was used as a 1–dimensional input grid with Gaussian filters. Specifically, each input neuron was located at coordinate 5, 15, 25,..., 95 in a arbitrary 1D space. The location of the neuron was the mean of the Gaussian filter, and all Gaussian filters had a standard deviation of 30. In each simulated trial, the location of one of the input neurons was randomly selected and a current of 70 mV was injected through the Gaussian filter. Because the Gaussian filters overlap, surrounding neurons also received current, but to a lesser extend based on the Gaussian filter. The exact timing of the injected current (and trial) is shown in Algorithm 1. Because the pre- and postsynaptic neuron layers were fully connected, the current was propagated to the two postsynaptic neurons according to the connection weights. The postsynaptic neuron with the most activation was selected as the* winner* and constituted the model response.

All plastic connections were initially random, and the network needed to learn to associate the first 5 presynaptic neurons with the first postsynaptic neuron and the last five presynaptic neurons with the second postsynaptic neuron using reinforcement learning. For example, if the first presynaptic neuron had received the most current and the winner was the first postsynaptic neuron, positive feedback was provided (in the form of dopamine release). In contrast, if the seventh presynaptic neuron had received the most current and the first postsynaptic neuron was the winner, negative feedback was provided (in the form of a dip in dopamine).

The simulation methodology is described in Algorithm 1. It should be noted that the simulation is exactly the same for both tripartite and bipartite networks. For example, if the first presynaptic neuron receives the current ‘I' as input in the first trial of the tripartite network, then the same neuron will receive the same amount of current in the first trial of the bipartite network. Also, the initial weights were exactly the same for the two networks.

### 3.2. Results

In this section, first, we present the results of implementing one single synapse. Next, the classification results of the bipartite and tripartite networks are provided.

*Synapse*. To compare tripartite synapses with bipartite synapses, we simulated the injection of a 70*mv* current to the presynaptic neuron for 1000*ms*. Then, we studied the changes in the voltage and spikes of neurons in both bipartite and tripartite synapses.

**Figure 6:**
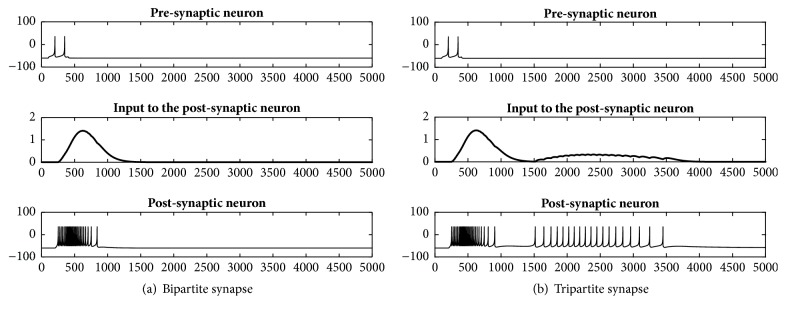
Behavior of neurons in a bipartite synapse (a) and tripartite synapse (b). The top and bottom panels show spikes in the presynaptic and postsynaptic neurons (respectively), while the middle panels show the input to the postsynaptic neuron.

The result of implementing a bipartite synapse, which consist of presynaptic and postsynaptic neurons, is presented in Figure 6.  Figure 6(a) shows from top to bottom the spikes of the presynaptic neuron, the output of the presynaptic neuron, and the spikes of the postsynaptic neuron.  Figure 6(b) presents the same results for the tripartite synapse introduced in Figure 4. The results show that adding an astrocyte in a tripartite synapse results in additional spikes after regular spikes (from the bipartite synapse) have ended in the postsynaptic neuron.

**Figure 7:**
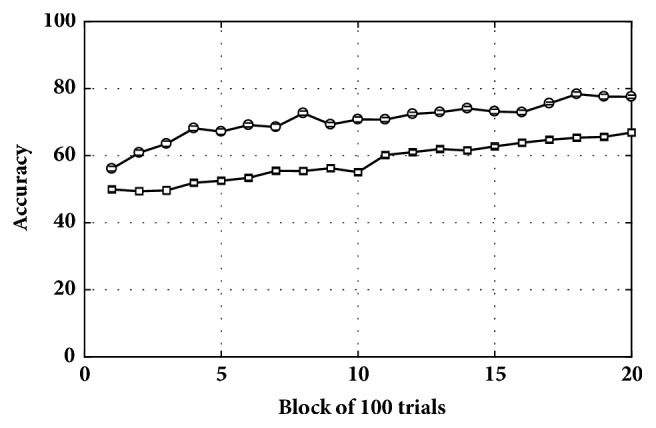
Classification accuracy for the tripartite (top) and bipartite (bottom) networks in a non-noisy condition.

*Networks*.  Figure 7 presents the accuracy results of classifying randomly selected inputs in a non-noisy condition (following Algorithm 1). As can be seen in the figure, the tripartite network was more accurate in classifying stimuli throughout learning. Final accuracy of the tripartite network was 77% (compared with 66% for the bipartite network). Hence, the performance of the tripartite network was superior in a noiseless environment.

**Figure 8:**
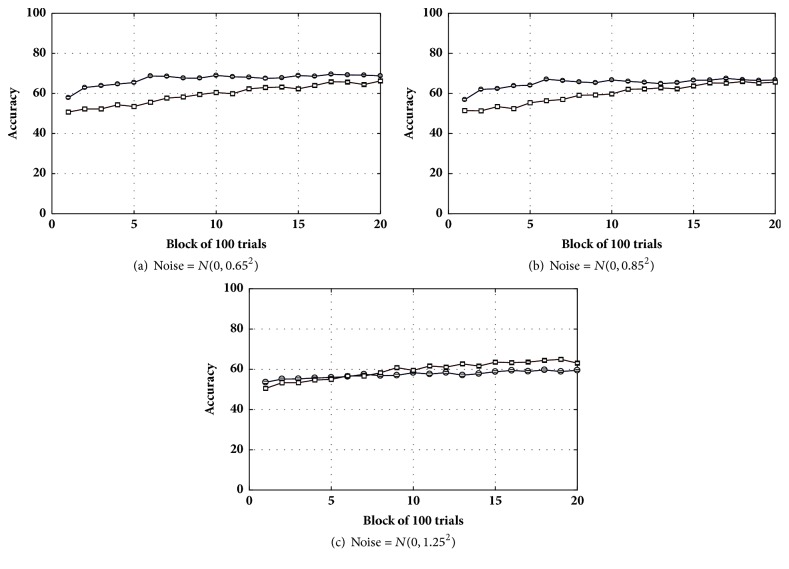
Accuracy in the classification task for the tripartite (circle) and bipartite (square) networks in noisy conditions.

Next, a small amount of noise, *N*(0,0.65^2^), was added to the voltage of the neurons.  Figure 8 shows that the tripartite network was less robust to noise in comparison to the bipartite network. The drop in accuracy caused by the added noise was larger in the tripartite network when compared to the bipartite network [66% to 66% (bipartite) versus 77% to 69% (tripartite); see Figure 8(a)]. Adding moderate noise *N*(0,0.85^2^) (Figure 8(b)), however, reduced the accuracy difference between the networks (65% versus 67% for the bipartite and tripartite networks, respectively). Finally, the accuracy difference all but disappeared with higher levels of noise *N*(0,1.25^2^) (Figure 8(c)). As can be seen in all three panels of Figure 8, the bipartite network was more robust and not much affected by the noisy conditions.

### 3.3. Discussion

The results presented in this section provide an answer to the question that was first asked: Are astrocytes capable of enhancing the performance of a neural network? The answer is ‘yes' (in the noiseless environment), although this result clearly does not mean that the tripartite network always work better than the bipartite network. To be more specific, our goal here was not to show that tripartite networks had an advantage over bipartite networks for all parameter values in all conditions. We only tried to show that astrocytes can be considered as a candidate for improving the performance of a neural network in specific conditions, and the role of astrocytes in improving the performance of a neural network is plausible. Further, we showed that the effect of astrocytes is to increase the length of activation (or number of spikes) in postsynaptic neurons.

## 4. General Discussion and Future Work

In this research we tried to answer the following questions:

**Is there a potential role for astrocytes in enhancing the performance of a neural network?**

The answer is yes, the computational result in this research suggest that there are conditions in which astrocytes improve synaptic plasticity and the performance of a neural network.

**Is reinforcement learning a good candidate for adjusting synaptic weights of tripartite networks? **The answer is yes, as shown in Figure 7, the tripartite network reaches the accuracy of more than 75% in classifying input stimuli. This suggests that reinforcement learning is successful in adjusting the synaptic weights.

**Are tripartite networks more robust to noise in comparison to bipartite networks?** Figure 8 shows the opposite. Injecting a small amount of noise to the voltage of neurons produced a dramatic drop in the accuracy of categorization in the tripartite network. In contrast, a bipartite network with the same parameters and noise almost kept the same performance.

### 4.1. Future Work

This research opens up possibilities for many future directions. First, by having a simple biologically realistic dynamical model of astrocytes, different theories about the roles of astrocytes can be tested more easily. For example, research in physiology shows that the number of astrocytes increases in neurodegenerative diseases [43, 44]. To explore how this increase would affect synaptic plasticity, spikes, and more generally the behavior of the network, one can implement a tripartite network with numerous astrocytes and test if the predicted behavior of the computational model matches the symptoms of these diseases. Second, more realistic models of tripartite networks can be developed. For example, some studies show that astrocytes also form a network and communicate through calcium waves [9, 45]. This calcium signaling in astrocytes is controlled by synaptically evoked neurotransmitters such as ATP, GABA, and glutamate [9, 46]. Astrocytes can also release these neurotransmitters into the synaptic cleft, a phenomenon called* gliotransmission* [9, 17, 46, 47]. As we learn more about gliotransmission, these additional processes can also be added to the tripartite network model to obtain more physiologically accurate results. A third possibility is related to artificial intelligence. In the past few years, very simple models of astrocytes were successfully added to artificial neural networks [24, 33, 34, 48]. The astrocyte model proposed in this research could provide new insights on designing more biologically accurate models of artificial astrocytes in artificial neural networks. Overall, it is our hope that providing a simple astrocyte model to the research community will contribute to increasing research about the roles of these cells in information processing.

## Figures and Tables

**Algorithm 1:**
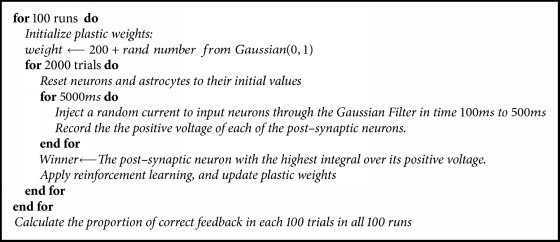
The working network.

**Table 1:** Parameter values used to model neurons and astrocytes based on the Izhikevich simple model of neurons. The neuron parameters represent a pyramidal neuron in neocortex [27].

	*C*	*v*_*r*_	*v*_*t*_	*k*	*a*	*b*	*c*	*d*	*v*_*peak*_
Neuron	100	-60	-40	0.7	0.03	-2	-50	100	35

Astrocyte	6	-70	1.429164 × 10^3^	2.77 × 10^−5^	0.03	−6.5 × 10^−4^	-50	100	35

**Table 2:** Parameter values used to implement reinforcement learning.

Parameters	Values
*θ*_*NMDA*_	1.5 × 10^2^

*θ*_*AMPA*_	5 × 10^2^

*η*	7.5 × 10^−2^

*α*_*w*_	5 × 10^−10^

*β*_*w*_	2 × 10^−10^

*γ*_*w*_	5 × 3^−13^

*w*_*max*_	2 × 10^3^

*non* − *plastic* *w*	7.1 × 10^−1^

## Data Availability

The data used to support the findings of this study are included within the article.
